# Case Series: Vestibular Migraines in Fragile X Premutation Carriers

**DOI:** 10.3390/jcm13020504

**Published:** 2024-01-16

**Authors:** YeEun Tak, Flora Tassone, Randi J. Hagerman

**Affiliations:** 1 University of California Davis School of Medicine, Sacramento Campus, Sacramento, CA 95817, USA; ytak@ucdavis.edu (Y.T.); ftassone@ucdavis.edu (F.T.); 2 Medical Investigation of Neurodevelopmental Disorders (MIND) Institute, University of California Davis Health, Sacramento, CA 95616, USA; 3 Department of Biochemistry and Molecular Medicine, University of California Davis, Sacramento, CA 95817, USA; 4 Department of Pediatrics, University of California Davis Health, Sacramento, CA 95817, USA

**Keywords:** vestibular migraine, vertigo, migraine, fragile X syndrome, fragile X premutation, fragile X-associated tremor/ataxia syndrome

## Abstract

Background: Vestibular migraine (VM) is one of the most common causes of recurrent vertigo and presents with a history of spontaneous or positional vertigo with a history of migraine headaches. While research has identified a high prevalence of migraine headaches and vestibular deficits among fragile X premutation carriers, there has been no discussion about VM within this population. Objective: This case series and review seeks to describe the clinical characteristics and pathophysiology of VM among individuals with the fragile X premutation. We also seek to discuss treatment and future steps in addressing VM in this population. Methods: A review of the literature regarding vestibular migraine and presentation of migraine headaches and vestibular deficits among premutation carriers was performed. A detailed clinical history of migraine headaches and vertigo was obtained from three patients with the fragile X premutation seen by the senior author (RJH). Results: All three cases first developed symptoms of migraine headaches earlier in life, with the development of VM near menopause. Two of the three cases developed progressive balance issues following the development of VM. All three cases found that their VM episodes were improved or resolved with pharmacological and/or lifestyle interventions. Conclusions: It is important to recognize VM among premutation carriers because beneficial treatments are available. Future studies are needed regarding the prevalence of VM and the relationship to subsequent FXTAS. The pathophysiology of VM remains uncertain but possibilities include mitochondrial abnormalities, cranial nerve VIII toxicity secondary to neurotoxic protein accumulation, and calcitonin gene-related peptide (CGRP) signaling dysfunction due to altered levels of fragile X messenger ribonucleoprotein (FMRP).

## 1. Introduction

Vestibular migraine (VM) presents with spontaneous or positional vertigo (usually with head motion, visually induced dizziness, dizziness with nausea) and is associated with a history of migraine headaches and migraine-associated symptoms [1]. It is one of the most common causes of recurrent vertigo, with a 1% prevalence in the general population and 10–20% of individuals presenting to headache specialty centers [2]. The diagnostic criteria for VM can be seen in Table 1.

### 1.1. History and Pathophysiology of VM

The term “vestibular migraine” was officially defined by the Barany Society and the International Headache Society (IHS) in 2012 [3]. Previously co-occurring presentations of migraine and vertigo had been referred to broadly as migraine-associated vertigo, migrainous vertigo, benign recurrent vertigo, etc. As opposed to generalized dizziness associated with migraine episodes, VM was indicated specifically as a vestibular manifestation of migraine. These vestibular symptoms are defined by the Barany Society’s classification of vestibular symptoms, which includes the definition of vertigo (internal: a false sense of self-motion vs. external: a false sense of surrounding movement) and different types of vertigo such as spontaneous, positional, visually induced, and head-motion-induced [4]. While VM occurs at any age, it is more prevalent among females and is often present throughout families. A common pattern of presentation involves migraine headaches at an earlier age, then development of vestibular migraine later in life [5]. Among women, migraine episodes seem to become vertigo attacks near menopause.

The etiology of VM is uncertain, but it is currently thought to be a crossover between genetic, neurochemical, and inflammatory causes. Studies have shown evidence for an autosomal dominant inheritance pattern of VM, and there has been a locus for familial VM identified on chromosomes 22q12 and 5q35 but no specific genetic defects [6,7]. The pathophysiology of migraine involves neurotransmitters such as calcitonin gene-related peptide (CGRP), serotonin, norepinephrine, and dopamine, which can also impact the activity of central and peripheral vestibular neurons and lead to the overall presentation of VM [2]. A notable mechanism of migraine headaches is inflammation of intracranial vessels, which results from serotonin-induced plasma extravasation in the dura mater. A study has shown this extravasation also occurs in the inner ear, and can lead to the development of vestibular symptoms in the setting of a migraine [8]. An additional etiology of VM is ion channel dysfunction in the inner ear. This is a plausible mechanism, as there are familial disorders involving migraine and vertigo that are caused by a genetic defect in a voltage-gated calcium channel (i.e., familial hemiplegic migraine and episodic ataxia type 2); however, no genetic defect has been identified for VM [9].

### 1.2. Fragile X Premutation Carriers

Individuals who are premutation (55 to 200 CGG repeats) carriers of the fragile X messenger ribonucleoprotein 1 gene (*FMR1)* are relatively common in the general population. The premutation is detected in approximately 1:130–250 females and 1:250–810 males [10,11]. Those with the fragile X premutation suffer from elevated levels of *FMR1*-mRNA, which causes neurotoxicity through sequestration of proteins that are important for neuronal function. This leads to oxidative stress, mitochondrial dysfunction, and DNA damage repair mechanisms. Repeat-associated non-AUG (RAN) translation producing FMR1polyG has also been found to occur in premutation carriers [12,13]. With aging, intranuclear inclusions (characterized as eosinophilic, ubiquitin-positive, and tau-negative) can form in neurons and astrocytes caused by the elevated levels of mRNA combined with misfolded proteins and ubiquitin-labeled proteins that are sequestered into the inclusions. These inclusions develop throughout the central and peripheral nervous system and in organs throughout the body [13,14]. As premutation carriers age, approximately 40–85% of males and 16% of females develop tremor and/or ataxia and are diagnosed with fragile X-associated tremor/ataxia syndrome (FXTAS). This is a neurodegenerative disorder which can also include neuropathy and white matter hyperintensities (especially in the middle cerebellar peduncles (MCPs), known as the MCP sign), central nervous system (CNS) atrophy, and progressive cognitive decline [15]. Clinically, premutation carriers can have a wide variety of symptoms and disease presentations, and any of these problems are collectively referred to as fragile X premutation-associated conditions (FXPAC) [15]. In addition to FXTAS, fragile X-associated primary ovarian insufficiency (FXPOI), meaning menopause before age 40, and fragile X-associated neuropsychiatric disorders (FXAND) including depression and anxiety are common diagnoses under FXPAC [15].

Pathophysiological changes such as mitochondrial dysfunction and calcium dysregulation can be present in premutation carriers long before the development of FXTAS [16,17,18]. The consequences of these changes can lead to GABA and glutamate dysregulation, symptoms of chronic pain and increased stress, and chronic illnesses such as autoimmune diseases and migraines [19,20].

### 1.3. Migraine and Vestibular Symptoms in Fragile X Premutation Carriers

Mitochondrial function abnormalities have been reported in premutation carriers both with and without FXTAS. An inverse relationship has been identified between mitochondrial protein levels and the number of CGG repeats in the *FMR1* premutation range [17]. It is thought that many of the symptoms experienced by premutation carriers can be attributed to mitochondrial dysfunction, and one clinical diagnosis of interest is migraine. Even migraineurs without a determined mitochondrial disorder exhibit dysfunction in mitochondrial oxidative phosphorylation [21,22]. A study by Au et al. [20] in 2013 identified an increased prevalence of migraine headaches among adult fragile X premutation carriers. The prevalence of migraine headaches was 54.2% in female *FMR1* premutation carriers vs. 25.3% in female controls, and 26.79% in male premutation carriers vs. 15.5% in male controls [20]. The proposed mechanism was related to mitochondrial dysfunction related to the premutation and worsening RNA toxicity with advanced age. The female premutation carriers also report increased symptoms of chronic pain, with high rates of allodynia, peripheral neuropathy, and migraine headaches [23].

In addition to migraine headaches, vestibular complaints have been found to be common among females with an *FMR1* premutation, with higher rates of dizziness reported among premutation mothers of children with fragile X syndrome (FXS) as well as in daughters of men with FXTAS compared with controls [24,25,26]. A study by Schneider et al. [27] observed deficits in prepulse inhibition (PPI, a measure of sensorimotor gating) among male patients with FXTAS. This was associated with hearing loss and a negative impact of RNA toxicity in the pons, basal ganglia, and cranial nerve VIII (CN VIII) [27]. Hearing loss was reported at a statistically higher rate among individuals with FXTAS (50%) compared with the general elderly population (30%) [28].

Balance deficits are a significant issue faced by premutation carriers with and without FXTAS. However, FXTAS patients face greater issues with vestibular balance, and they have white matter disease that begins in the cerebellum and basal ganglia caused by the RNA toxicity of elevated mRNA levels [29]. Cerebellar degeneration leads to issues with vestibular control of balance because the cerebellar vermis plays an important role in postural control [30]. Neuroimaging studies have shown decreased cerebellar vermis volume among FXTAS patients [31,32,33]. The inferior cerebellar peduncles (ICPs) transfer information between the cerebellum and vestibular nuclei for vestibular sensory processing [30]. Although the middle cerebellar peduncles (MCPs) are typically affected with white matter disease in FXTAS, there is involvement throughout the cerebellum and the pons. The pontine nuclei and MCPs are reported areas of neurodegeneration in FXTAS, and these areas are known for controlling planned movements [15,29,33,34].

While research has identified a high prevalence of migraine headaches and vestibular deficits among premutation carriers, there has been no discussion of VM within this population. The following case series describes the experiences of three female patients with the premutation and VM seen by the senior author (RJH) and seeks to characterize the presence of VM among individuals who are premutation carriers.

## 2. Materials and Methods

### 2.1. Subjects

Three female carriers of the *FMR1* premutation were evaluated. All subjects were seen as a part of a family study of probands diagnosed with fragile X syndrome (FXS) or part of an adult study of individuals who are premutation carriers with and without FXTAS. No subject was referred to our clinic for headache- or vestibular-related problems. All subjects were seen by the senior author (RJH). All subjects signed an informed consent. A standardized medical history and physical examination were performed by a physician (RJH). The medical history touched on specific questions regarding migraine history, development of vertigo symptoms, work-up for vestibular migraine, and current presentation. All subjects underwent confirmatory *FMR1* DNA testing either at our facility or an outside facility. *FMR1* molecular measures included CGG repeat number.

### 2.2. Case 1

Case 1 is a 78-year-old female with a history of the premutation (107 CGG repeats), migraine headaches, and vertigo. She has a younger sister with a premutation and younger brother and son with FXS. She began having episodes of migraine headaches with aura in her 40s–50s. In 2018, she underwent earwax removal at her primary care provider’s (PCP’s) clinic and began experiencing severe symptoms of dizziness shortly after. The patient was referred to a neurophysiotherapist, who initially diagnosed her with positional vertigo based on her history of spinning dizziness, exacerbated by laying down and head turning, and a clinical evaluation. She was recommended home exercises, which mildly improved her symptoms but her episodes of vertigo persisted. In 2019, the patient underwent a vacuum earwax removal with an otolaryngologist (also known as an ENT), and had an episode of vertigo during this visit. In 2020, a head magnetic resonance imaging (MRI) was performed due to concerns of unilateral hearing loss with tinnitus, which did not show any focal abnormalities. It did, however, reveal diffuse cerebral atrophy and periventricular white matter disease. A follow-up visit in 2021 with the ENT specialist involved repeat vacuum earwax removal, and after more extensive history taking, she was diagnosed with VM. The patient’s vertigo episodes persisted, and her balance progressively worsened as well. Her younger sister had similar symptoms of vertigo and balance issues. The patient’s primary health care provider (PCP) decided to start oral verapamil hydrochloride SR 240 mg once per day, given its benefits in the setting of migraine-related vertigo. The patient reported improvement in the episodes of vertigo, but her balance problems progressed, and she now has a shuffling gait. She has been recommended for further work-up for FXTAS given the progression of balance issues and prior MRI findings consistent with individuals with FXTAS.

### 2.3. Case 2

Case 2 is a 59-year-old female with a history of the premutation (69 CGG repeats), migraine headaches, and vertigo. She has two sons with FXS. The patient’s mother is also a premutation carrier and had a history of migraine. As a child, the patient had frequent episodes of motion sickness. She began having occasional episodes of migraine headaches in her 20s (1–2 x/year), with an episode of an ocular migraine in her 40s, causing eye pain, photophobia, and vision changes. The frequency of migraine headache episodes gradually subsided over time. The patient then began having episodes of dizziness in her 40s, which would last for about a day and occur every few months. These episodes involved a sensation of spinning with movement and head turning. They were often mild, but she had her worst vertigo episode, persisting for three days, when she was 51. Her ENT diagnosed her with vestibular migraine following clinical evaluation, and recommended septoplasty as they felt the vertigo episodes were exacerbated by a deviated septum causing mild hypoxia while sleeping. The patient subsequently underwent a septoplasty a few months later. At that time, she was also prescribed oral verapamil 200 mg ER once per day. Following septoplasty and after starting verapamil, the patient found that her vertigo symptoms had improved. Additionally, she began eating a Paleolithic diet, which she reported improved her allergies, and she has not had a recurrent episode of vertigo since.

### 2.4. Case 3

Case 3 is a 63-year-old female with a history of the premutation (81 CGG repeats), migraine headaches, and vertigo. She has two sons with FXS, and her father passed away with FXTAS. She began having migraine episodes in her late 30s/early 40s and felt it overlapped with the onset of her menstrual cycles. These episodes would occur once or sometimes twice a month and were associated with photophobia and vision changes. She was eventually diagnosed with ocular migraine by her ophthalmologist. The patient was started on a sublingual triptan and found that her migraine episodes improved. In addition to migraine, the patient developed intermittent vertigo episodes that started around age 50. She described it as a “wall tilting” and “room spinning” sensation that would worsen with movement and head turning. The patient had these episodes biannually, and each episode would persist for about a few hours. She was diagnosed with vestibular migraine but did not take medications for preventative or abortive treatment as her episodes were often resolved by sleeping. The patient also reported progressive balance problems with onset in her late 50s, which has caused her to become unable to walk, bike, or drive in a straight line. The patient has developed a mild tremor while holding eating utensils in her hands but reported that her tremors are not as severe as her father’s were. She will be undergoing an evaluation soon for FXTAS with a head MRI.

## 3. Discussion

### 3.1. VM and FXTAS

All three patients reported in our case series are female, and migraine episodes manifested earlier in life, with the development of vestibular symptoms near menopause—which are common characteristics of individuals with VM [2]. Based on the diagnostic criteria for VM by Lempert et al. [3], our patients would fall under the “probable VM” criteria, as they often presented to their ENT or primary doctor with a history of migraine headaches and with a new onset of vertigo episodes. These patients meet the diagnostic criteria for benign paroxysmal positional vertigo (BPPV) based on the description of the positional dizziness episodes; however, the long duration of the attacks (>1 min) and the history of migraine headaches suggested that the episodes were of another vestibular disorder [35].

The fragile X premutation is associated with the sequestration of proteins important for neuronal function, and there is enhanced oxidative stress and mitochondrial dysfunction in premutation carriers, especially in those with FXTAS [36]. Other manifestations of CN VIII toxicity in FXTAS include hearing loss, tinnitus, and imbalance, which are common [15,27,37]. A study by Mitchell et al. [38] revealed that FMRP may be a modulator of calcitonin gene-related peptide (CGRP) signaling in nociceptive sensitization and chronic pain. CGRP signaling plays an important role in hearing and balance and the development of migraine [39]; therefore, altered levels of FMRP in premutation carriers could contribute to further dysfunction of CN VIII and increased risk of developing VM. Furthermore, the pons and cerebellum are areas of the CNS with significant involvement in white matter disease and atrophy in FXTAS [36]. Case 1’s MRI findings of white matter disease and cerebral atrophy were consistent with findings of FXTAS. It is interesting that both Case 1 and Case 3 developed VM first, which gradually progressed to more significant balance problems and symptoms of FXTAS. Case 2 was not noted to have these progressive balance problems; however, she is younger than the other two cases and will have to be further monitored for development of FXTAS symptoms. Further study is recommended to determine the prevalence of VM among premutation carriers and whether it could be a precursor to FXTAS.

### 3.2. VM Treatment

Historically, treatment for VM has involved lifestyle changes, medications, physical therapy, and acupuncture [40]. Abortive treatment of acute VM has involved non-steroidal anti-inflammatory drugs (NSAIDs), paracetamol, antiemetics, and triptans. Medications recommended for preventative treatment of VM have included antiepileptics, calcium channel blockers, beta-blockers, and antidepressants (i.e., tricyclics, serotonin, and norepinephrine reuptake inhibitors). In 2021, a meta-analysis [41] provided treatment guidelines for VM. The strongest recommendations for preventative treatment with the best efficacy and least side effects were tricyclics (amitriptyline, nortriptyline), beta-blockers (propranolol), and calcium channel blockers (flunarizine, verapamil) [41].

Non-selective calcium channel blockers (CCBs) have typically been recommended for VM prophylaxis. Verapamil (a selective CCB) has also been used, and in a retrospective cohort study in 2021, three patients were treated with verapamil 120 mg twice a day, which provided a minimal/minor improvement of symptoms [42]. Case 1 and Case 2 found verapamil helpful for their episodes of VM, but the current literature indicates that verapamil is not as highly recommended for VM [41]. There is more evidence for the effectiveness of non-selective CCBs (flunarizine, cinnarizine, and lomerizine) in VM prophylaxis [41,43,44,45]. It is possible that pharmacological treatment, in addition to other modalities of treatment such as addressing secondary triggers via surgery or lifestyle changes, led to the improvement of our patients’ VM symptoms.

Lifestyle changes (increased sleep and exercise and avoidance of dietary triggers such as alcohol and caffeine) have been highly recommended to all patients with VM, and vestibular rehabilitation is recommended to patients refractory to or intolerant of pharmacological treatment [41,46]. Brevern and Lempert [47] in their 2020 review article suggest that lifestyle changes can be as effective as pharmacological measures, and medications should primarily be involved in the case of acute or frequent VM attacks. Case 1 initially elected for vestibular rehabilitation through home exercises to address her symptoms of vertigo, but did not find significant relief, so she subsequently took verapamil, which did provide relief. Case 2 was also helped by verapamil, and she found that a Paleolithic diet remarkably improved her allergies and has not had a further episode of VM since. A Paleolithic diet includes components that mirror the human diet during the Paleolithic era, such as fresh fruits, vegetables, lean meats, and fish [48]. This is consistent with the diet recommendations provided to patients with VM, which includes avoiding dietary triggers such as processed meats, alcohol, and aged cheeses [41,46]. There are also a number of clinical trials observing the benefits of a ketogenic diet (KD) in migraine prophylaxis, reducing attack frequency and intensity [49,50,51,52]. The mechanism of the ketogenic diet is thought to be related to the enhancement of mitochondrial metabolism and reduction in neural inflammation [50]. Therefore, a ketogenic diet could also be considered for VM prophylaxis for patients with the premutation.

## 4. Conclusions

VM has been determined to be the most common cause of recurrent vertigo, and while its presentation was previously unclear, it was established with diagnostic criteria by the Barany Society and the International Headache Society (IHS) in 2012 [3]. The pathophysiology of VM remains uncertain but possibilities include mitochondrial abnormalities, CN VIII toxicity secondary to neurotoxic protein accumulation, and CGRP signaling dysfunction due to altered levels of FMRP. It is notable that migraine headaches and vestibular problems are common clinical findings among individuals with the fragile X premutation [20,24,25,26]. A detailed case series of three women who are premutation carriers revealed symptoms characteristic of VM. This is the description characterizing the presence of VM among patients with the fragile X premutation. All three patients first experienced a history of migraine headaches prior to the development of vertigo near menopause. Two patients then progressed to developing symptoms of imbalance and/or tremor, characteristic of FXTAS. Two patients improved after treatment with verapamil, and one underwent septoplasty along with adopting a Paleolithic diet which proved effective. It seems that a combination of both medication and lifestyle changes was the most effective treatment for our patients with the premutation and VM.

A limitation of our study was its small sample size of patients, and we would recommend a larger study that determines the prevalence of VM in patients with the premutation, as well as what percentage of these patients progress to developing FXTAS. Another limitation was the inability to obtain more detailed neuro-otological data from outside providers. All the cases had been evaluated by an outside ENT or primary providers for their vestibular migraine symptoms, but we were not able to obtain outside records outlining the clinical work-up. Therefore, we sought to describe the history of the patients’ migraines and vertigo episodes in detail. Additionally, we would have liked to obtain a head MRI for our Case 3 patient, as it would have provided further information about her development of FXTAS.

## 5. Future Directions

It is important to recognize VM in premutation carriers because in our cases two are developing FXTAS. Further study is required to determine whether VM could be a precursor or harbinger of FXTAS among premutation carriers, and whether interventions can be initiated with the onset of VM symptoms to prevent further progression towards FXTAS. Premutation carriers with VM should be followed carefully over time, particularly those older than 60 and with higher CGG repeats in the premutation range. Given the high prevalence of hearing and vestibular problems among premutation carriers [20,24,25,26], further studies are recommended to identify the mechanisms of CN VIII toxicity in these individuals and how progressive auditory and balance issues can be mitigated. Future research should also determine the true prevalence of VM among individuals with the fragile X premutation. Regarding the development of progressive balance issues and FXTAS, there is no cure currently, but there is ongoing research to develop targeted gene therapies and clinical trials [15]. An open-label study utilizing allopregnanolone for men with FXTAS has been found to improve a limited number of symptoms associated with FXTAS [53]. Participants reported improved executive function, learning, and memory. Cannabidiol (CBD) is undergoing clinical trials for individuals with fragile X syndrome due to its anti-inflammatory and neuroprotective properties [54]. However, CBD treatment needs to be formally studied among premutation carriers, especially to address insomnia, pain symptoms, and anxiety and perhaps also FXTAS symptoms. Anavex 2–73 is an experimental small molecule that is thought to reduce oxidative stress and improve mitochondrial function. It is currently being considered for a treatment of FXTAS [54]. More studies are required to develop treatments to address mitochondrial dysfunction leading to the development of FXTAS in premutation carriers, and these treatments are also likely to help VM.

## Figures and Tables

**Table 1:** Diagnostic criteria for vestibular migraine [3].

Vestibular migraine: 1. At least 5 episodes with vestibular symptoms of moderate or severe intensity, lasting − 5 min to 72 h. 2. Current or previous history of migraine with or without aura according to the International Classification of Headache Disorders (ICHD-3). 3. One or more migraine features with at least 50% of the vestibular episodes: − Headache with at least 2 of the following characteristics: 1-sided location, pulsating quality, moderate or severe pain intensity, aggravation by routine physical activity. − Photophobia and phonophobia. − Visual aura. 4. Not better accounted for by another vestibular or ICHD diagnosis.
Probable vestibular migraine (not included in ICHD-3): 1. At least 5 episodes with vestibular symptoms of moderate or severe intensity, lasting − 5 min to 72 h. 2. Only 1 of the criteria B and C for vestibular migraine is fulfilled (migraine history or migraine features during the episode). 3. Not better accounted for by another vestibular or ICHD diagnosis.

## Data Availability

The original contributions presented in the study are included in the article, further inquiries can be directed to the corresponding author.
